# Metabolic Syndrome and Insulin Resistance in Schoolchildren From a Developing Country

**DOI:** 10.3389/fnut.2020.00031

**Published:** 2020-03-31

**Authors:** Rashmi Ranjan Das, Manaswini Mangaraj, Sandeep Kumar Panigrahi, Amit Kumar Satapathy, Samarendra Mahapatro, Partha Sarathi Ray

**Affiliations:** ^1^Department of Pediatrics and Biochemistry, AIIMS, Bhubaneswar, India; ^2^Department of Community Medicine, IMS and SUM Hospital, Siksha ‘O' Anusandhan deemed to be University, Bhubaneswar, India

**Keywords:** obesity, overweight, cross-sectional study, metabolic syndrome, HOMA-IR

## Abstract

**Background:** Overweight and obesity are prevalent in schoolchildren due to dietary habits and lack of exercise. These children are prone to metabolic syndrome (MS) and future risk of type 2 diabetes mellitus and cardiovascular diseases.

**Materials and Methods:** This cross-sectional study was conducted in Bhubaneswar City, Eastern India, among schoolchildren. Obesity and overweight were determined by the Indian Academy of Pediatrics guideline. Fasting venous blood samples were taken for insulin, blood glucose, and lipid levels measurement. Blood pressure was measured as per the protocol. The International Diabetic Federation (IDF) criteria for the definition of MS were followed. Insulin resistance was determined by a homeostatic model assessment (HOMA-IR).

**Results:** A total of 1,930 children were screened, of which 545 (28.2%) were overweight and obese. The male to female ratio was 1.27. The overall prevalence of MS was 21.8% (11% in 6 to ≤10 years old and 30.6% in 11 to 16 years old). A history of cardiovascular disease, diabetes, obesity, and hypertension in the family was present in 42.7%. Acanthosis nigricans was present in 46.4%. A history of exclusive breast feeding for 6 months was present in 68.1%. The mean HOMA-IR in children with MS was 5.46 compared to 2.18 in those without MS (insulin resistance was more common in children with MS).

**Conclusions:** The present study found a higher prevalence of MS and insulin resistance in schoolchildren from Eastern India who are overweight/obese.

## Introduction

Schoolchildren nowadays have increased prevalence of overweight and obesity due to lack of exercise and improper dietary habits. Besides the high-income countries, the prevalence is increasing in low- and middle-income countries. In 2016, as per the World Health Organization (WHO), 41 million children under 5 years old were overweight or obese (1). Nearly 50% of them were under 5 years old and lived in Asia. Over 340 million children and adolescents over 5 years old were overweight or obese (1). The prevalence of overweight or obesity in the latter age group has increased dramatically from 4% (in 1975) to >18% (in 2016) with a nearly equal proportion among both sexes (18% in girls and 19% in boys) (1). Regarding obesity, the prevalence in this age group was <1% in 1975, but in 2016 it has increased to >124 million (6% of girls and 8% of boys) (1).

In a systematic review on overweight and obesity in the Indian subcontinent using the International Obesity Task Force (IOTF)–Cole et al. criteria, the pooled prevalence of obesity in children under 5 years old was <2%, while in those over 5 years old (not adolescents) it was 2 to 8% (2, 3). Among the adolescents, the prevalence of overweight was 3.0 to 24.7%, and obesity was 1.5 to 14% (2, 3).

Overweight and obesity are linked to more deaths worldwide than underweight. Globally, there are more people who are obese than underweight—this occurs in every region except in parts of sub-Saharan Africa and Asia. Children with obesity are prone to metabolic syndrome (MS) and future risk of type 2 diabetes mellitus and cardiovascular diseases. Although there are many reports on children with obesity, there is paucity of data on children affected with MS from settings in developing countries. Our primary objective was to determine the prevalence of MS in schoolchildren with overweight/obesity. Our secondary objectives was to determine the prevalence of overweight/obesity and insulin resistance in these children.

## Materials and Methods

This school-based cross-sectional study was conducted in Bhubaneswar City, Eastern India, over a 1-year period (April 2017 to March 2018). Schoolchildren 6 to 16 years of age were included. The exclusion criteria were major medical illness including diabetes mellitus, physical deformity, chronic medication use that can cause overweight/obesity/metabolic syndrome, and unwillingness to participate in the study. Cluster random sampling method was used with the cluster being schools. A trained research officer made school visits under the supervision of the principal investigator. After obtaining permission from the school principal, the participant information sheets and informed consent documents were distributed to the schoolchildren to be read in detail by the parents at home and brought back on the next day. Clarifications from the parents regarding the study, if any, were given through telephonic contact with the research team.

Anthropometric measurements including height, weight, waist, and hip circumference were obtained. Height (taken with the schoolchildren not wearing their shoes) was measured with a stadiometer to the nearest centimeter (cm). Weight was measured to the nearest 100 g, with the schoolchildren wearing light clothing but without shoes. Waist circumference (WC, cm) was measured, with the schoolchildren in a standing position, using a non-stretchable measuring tape at midpoint between the costal margin and iliac crest in the mid-axillary line at the end of expiration. Hip circumference (cm) was measured at the prominence of the buttocks. Blood pressure (BP) was measured with a standardized sphygmomanometer with appropriately sized cuffs. BP was measured in the right arm, in sitting position, three times (at 0-, 5-, and 30-min intervals), and the average of the readings was taken. Body mass index (BMI) was calculated (kg/m^2^) as per the standard formula and classified into overweight or obesity as described previously (4). Fasting venous blood samples of eligible children were taken for insulin, blood glucose, triglyceride, and low density lipoprotein (LDL) and high density lipoprotein (HDL) cholesterol measurement, and the abnormalities were defined as per the data published in Indian children previously (5, 6). Plasma glucose was obtained by the glucose oxidize technique, and serum lipids were measured with a Beckman Coulter (AU5800 Clinical Chemistry Analyzer, Danaher Corporation, California, USA). Serum insulin level was determined with a Beckman Coulter (Access 2 Immunoassay System, Danaher Corporation, California, USA). At the same time, data on age, gender, birth weight, exclusive breastfeeding (EBF) in the first 6 months, and family history of cardiovascular disease (CVD)/non-communicable diseases (NCD)/type 2 diabetes/ hypertension in the family (first- and second-degree relatives) were recorded. Waist and hip circumference cutoffs were defined as per the previously published guideline in Indian children (7). Hypertension was defined as per the international guideline published by the American Academy of Pediatrics (8). Physical examination was done for the presence of acanthosis nigricans (on the neck, axillae, and skin folds) and hepatomegaly.

The International Diabetic Federation (IDF) criteria for the definition of metabolic syndrome were followed in children >10 years of age (9). The IDF criteria defines MS as the presence of obesity (WC ≥ 90th centile or adult cutoff if lower) plus two or more risk factors [fasting blood glucose (FBG) ≥ 100 mg/dl or type 2 diabetes mellitus, elevated blood pressure defined as systolic BP (SBP) ≥ 130 mmHg or diastolic BP (DBP) ≥ 85 mmHg, and dyslipidemia defined as triglyceride (TG) ≥ 150 mg/dl or HDL <40 mg/dl]. In children who were overweight (BMI ≥ 85th centile), we applied IDF obesity criteria if there was central obesity (WC ≥ 90th centile). In children aged ≤10 years old, the criteria adopted by Boney *et al*. among children in the USA were used (10). Boney et al. defined MS as the presence of two or more of the following four criteria: obesity (BMI ≥ 85th centile), hypertension (SBP or DBP ≥ 95th centile for age), dyslipidemia TG ≥ 95th centile or HDL <5th centile for age), and glucose intolerance (FBG >110 mg/dl or a 2-h postprandial glucose level of >140 mg/dl after a standard mixed meal).

Insulin resistance was determined by a homeostatic model assessment (HOMA-IR) (11). HOMA-IR was calculated by multiplying the fasting plasma insulin level (mIU/L) with the fasting plasma glucose level (mmol/L) and then dividing by 22.5 (11). Insulin resistance was defined as a HOMA-IR score ≥ 3.16. Hyperinsulinemia was defined as a fasting insulin level >17 mIU/L (12, 13).

### Sample Size Calculation

Two-stage cluster random sampling method was used, with the cluster being schools. The number of public and private schools was selected randomly and proportionate to the number present in the city. Considering the mean prevalence of overweight/obesity among schoolchildren to be 28% and prevalence of MS among children with overweight/obesity to be 20% (from different studies), it was calculated that the prevalence of MS in normal schoolchildren would be 5.6% (0.28 multiplied by 0.20). With an absolute error of 2%, it was estimated that a total of 508 samples would be required for the study if conducted using simple random sample (14). Considering cluster random sampling design that was selected for the study, a design effect of four was used to arrive at a final minimum sample size of 2,032. An additional 10% sample was used considering the chances of non-responders, parents not giving consent, or chances of error in blood sample collection, etc. Thus, a total of 2,235 minimum number of samples were taken as the final sample size for screening MS.

### Statistical Analysis

Data were analyzed using the statistical software STATA 13.0. Descriptive data were presented as number and proportions. Independent samples t-test was applied to compare the means, and the proportions were compared using chi-square test. Linear regression analysis was done using variables that were found to be significant on bivariate analysis and also those possible to have an influence on HOMA-IR. A *p* < 0.05 was considered as statistically significant.

## Results

**Figure 1:**
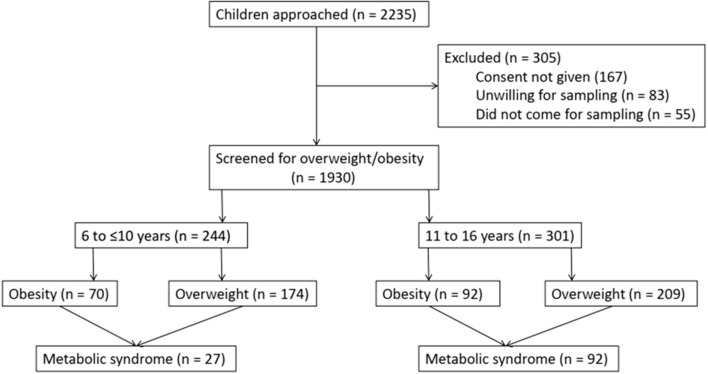
Flow of this study's children.

A total of 2,235 children were screened and 305 (13.6%) children were excluded because of non-consent or unwillingness for blood sampling. A total of 1,930 children were screened for overweight/obesity based on BMI (Figure 1). Of the 1,930 children, 602 (31.2%) were from government schools and 1,328 (68.8%) were from private schools. A total of 545 children (28.2%) were found to be overweight/obese [overweight = 383 (19.83%), obese = 162 (8.3%)]. The prevalence of overweight/obesity in schoolchildren 6 to 16 years of age in government schools was 69/602 (11.5%) and in private school was 476/1,328 (35.8%). The male to female ratio was 1.27 (305/240). Of the 545 children, 244 were 6 to ≤10 years old [obesity = 70 (28.7%), overweight = 174 (71.3%)], and 301 were 11 to 16 years old [obesity = 92 (30.6%), overweight = 209 (69.4%)]. The prevalence of central obesity (WC >90th centile) was 13.6% in children who were overweight (*n* = 52) and 68.4% in children who were obese (*n* = 111). The detailed analysis is provided in Table 1.

**Table 1:** Anthropometric, clinical, and biochemical parameters among the study population.

**Parameters**	**MS Present**		**MS absent**		***p*-value**
	**Mean**	**SEM**	**Mean**	**SEM**	
**Type of school**^***a***^					
Private	104	21.8%	372	78.2%	0.98
Government	15	21.7%	54	78.3%	
**Sex**^***a***^					
Male	59	19.3%	246	80.7%	0.11
Female	60	25.0%	180	75.0%	
**Age group**^***a***^					
6 to ≤10 years old	27	11.1%	217	88.9%	<0.01^*^
11–16 years old	92	30.6%	209	69.4%	
Age (years)	11.27	0.20	9.57	0.12	<0.01^*^
Weight (kg)	50.56	1.14	39.76	0.60	<0.01^*^
Height (cm)	145.10	1.21	136.91	0.72	<0.01^*^
**BMI (KG/M**^**2**^**)**	23.54	0.20	20.56	0.11	<0.01^*^
Blood pressure (mmHg)					
Systolic BP	121.98	0.54	113.16	4.50	0.3
Diastolic BP	80.05	0.43	71.29	0.29	<0.01^*^
Waist circumference (cm)	79.77	8.23	70.06	7.81	<0.01^*^
Waist circumference >90th centile^*a*^	106	89%	57	14%	<0.01^*^
Hip circumference (cm)	67.05	0.77	53.34	0.42	<0.01^*^
**Family history of NCD and CVD**^***a***^					
Present	65	27.9%	168	72.1%	<0.01^*^
Absent	54	17.3%	258	82.7%	
**EBF for 6 months**^***a***^					
Present	72	19.4%	299	80.6%	0.045^*^
Absent	47	27.0%	127	73.0%	
**Biochemical parameters**					
FBG (mg/dl)	99.34	0.54	83.51	0.30	<0.01^*^
TG (mg/dl)	160.10	1.55	110.91	1.10	<0.01^*^
HDL (mg/dl)	35.80	0.29	44.11	0.15	<0.01^*^
LDL (mg/dl)	97.88	0.81	80.78	0.46	<0.01^*^
Insulin (mIU/L)	22.06	0.64	10.47	0.21	<0.01^*^
HOMA_IR	5.47	0.17	2.18	0.04	<0.01^*^
**Acanthosis nigricans**^***a***^					
Present	107	42.5%	145	57.5%	<0.01^*^
Absent	12	4.1%	281	95.9%	
**Hepatomegaly**^***a***^					
Present	44	77.2%	13	22.8%	<0.01^*^
Absent	75	15.4%	413	84.6%	

*^1^Indicate numbers with proportion*.

* *p-value < 0.05*.

*BMI, body mass index; FBG, fasting blood glucose; TG, triglyceride; HDL, high density lipoprotein; LDL, low density lipoprotein; SBP, systolic blood pressure; DBP, diastolic blood pressure; EBF, exclusive breastfeeding; HOMA-IR, indicates insulin resistance and calculated by multiplying insulin with fasting blood glucose; CVD, cardio-vascular disease; NCD, non-communicabledisease*.

Cardiovascular disease, diabetes, obesity, and hypertension in the family were present in 42.7%. Acanthosis nigricans was present in 46.4%. A history of EBF for 6 months was present in 68.1%. The liver was enlarged in 8.1%. The detailed analysis is provided in Table 1.

The overall prevalence of metabolic syndrome in overweight/obese children was 21.8% (11% in 6 to ≤10 years old and 30.6% in 11 to 16 years old). The prevalence of individual components/risk factors of MS is provided in Table 2. As can be seen, low HDL was the most common (22.6%) and hyperglycemia was the least common (12.8%) component/defining criteria for MS. Except for obesity, the risk factors were more common in girls than in boys. Only one adolescent had all the risk factors of MS.

**Table 2:** Prevalence of individual risk factors of the metabolic syndrome.

	Obesity^*^, % (95% CI)	**Hyperglycemia, % (95% CI)**	**Hypertriglyceridemia, % (95% CI)**	**Low HDL, % (95% CI)**	**Elevated BP, % (95% CI)**
Total	29.7% (28.2 – 30.6)	12.8% (11.8–16.2)	20.8% (19.2–21.6)	22.6% (21.3–23.4)	15.4% (13.5–18.8)
**Sex**					
Male	29.8% (27.8–30.7)	13.1% (12.3–15.7)	19.6% (18.7–21.4)	21.6% (20.5–22.1)	15.8% (14.3–17.2)
Female	29.2% (27.6–30.6)	12.6% (11.2–15.5)	21.4% (19.5–21.8)	23.1% (21.7–23.8)	14.7% (12.9–16.7)
**Age (Y)**					
6– <8	23.8% (21.6–25.2)	2.4% (1.9–4.7)	18.7% (16.9–20.4)	20.2% (18.7–21.9)	12.4% (10.8–13.6)
8– <10	31.1% (29.3–32.1)	5.2% (3.8–7.5)	19.9% (18.1–21.7)	22.3% (19.6–13.5)	16.2% (14.6–17.7)
10– <12	31.2% (30.2–31.9)	17.8% (16.7–19.1)	21.3% (19.5–22.4)	23.1% (20.8–24.4)	18.4% (16.8–20.1)
12– <14	31.1% (29.4–33.2)	19.2% (18.1–21.4)	21.9% (20.1–22.8)	23.5% (21.1–24.6)	18.9% (17.2–20.2)
14 −16	29.2% (27.8–30.7)	19.4% (17.7–21.5)	21.7% (19.8–22.4)	22.9% (20.9–24.2)	19.8% (18.1–21.2)
**BMI**					
Overweight	70.3% (68.6–71.4)	4.6% (3.2–5.3)	5.8% (5.1–7.1)	7.4% (6.2–8.1)	6.9% (5.9–8.2)
Obesity	29.7% (28.2–30.6)	28.3% (26.7–29.5)	34.5% (32.7–35.8)	35.1% (34.3–36.4)	27.2% (26.6–28.5)

* *Obesity was defined by two different definitions for MS in children 6 to ≤10 years (as BMI ≥85^th^ centile) and 11 to 16 years of age (as waist circumference >90th centile). As the data for both age groups have been clubbed together in this table, the obesity % (29.7%) mentioned here is based on BMI only (not to be read as “prevalence of obesity in children with overweight is 70.3%”). For prevalence of central obesity (waist circumference >90th centile), please refer to the “RESULTS” section and Table 1*.

*CI, confidence interval*.

A univariate analysis of each component of MS by potential predictive factors (age, gender, obesity, family history of CVD and NCD, and EBF at 6 months) was carried out. Subsequent multivariate models (Table 3) showed that female gender was not significantly associated in any of the components of MS. High BP was also not significantly associated with any of the variables (age, gender, family history of CVD and NCD, and EBF at 6 months). Central obesity (WC > 90th centile) was significantly associated with age and family history of CVD and NCD. High FBG was significantly associated with age and central obesity. High triglyceride was associated with age, obesity, and family history of CVD and NCD. Low HDL cholesterol was significantly associated with a family history of CVD and NCD and absent EBF at 6 months. In the case of high blood pressure, no variable was qualified for the multivariate analysis.

**Table 3:** Multivariate analysis [odds ratios (95% CI)] for components of MS.

**Variables**	**WC > 90th centile**	**High FBG**	**High triglyceride**	**Low HDL**	**High BP**
**Age**					
≤ 10 years old	-	-	-	-	-
>10 years old	**2.03 (1.56–2.14)**	**2.66 (1.48–4.92)**	**1.29 (1.03–2.04)**	1.12 (0.92–1.43)	1.82 (0.87−2.01)
**Gender**					
Male	-	-	-	-	-
Female	0.92 (0.85–1.32)	1.34 (0.89–1.77)	1.09 (0.91–1.23)	1.12 (0.76–1.52)	1.14 (0.88–1.3)
**Overweight**					
No	-	-	-	-	-
Yes	**49.0 (26.7–99.2)**	**3.28 (1.47–3.73)**	**2.28 (1.69–3.43)**	1.53 (0.81–2.01)	1.42 (0.99–2.06)
**Family History of NCD and CVD**					
Present	-	-	-	-	-
Absent	**1.46 (1.28–2.23)**	1.0 (0.81–1.13)	**1.89 (1.01–2.37)**	**2.43 (1.41–3.02)**	1.14 (0.77–1.96)
**EBF for 6 months**					
Present	-	-	-	-	-
Absent	1.08 (0.64–1.03)	1.19 (0.95–1.76)	0.83 (0.54–1.06)	**1.21 (1.05–1.68)**	1.12 (0.79–1.48)

*Significant associations (p < 0.05) are shown in bold*.

The mean HOMA-IR in children with MS was 5.46 (insulin resistance defined as HOMA-IR score ≥ 3.16) compared to 2.18 in those without MS. A linear regression analysis was done using variables that were found to be significant on bivariate analysis and also those possible to have an influence on HOMA-IR. The variables that were found to be significant were TG [RR 5.8 (95% CI 4.7–7.4)], waist circumference [RR 3.2 (95% CI 2.8–3.6)], diastolic BP [RR 3.5 (95% CI 3.1–3.9)], systolic BP [RR 2.9 (95% CI 2.4–3.5)], and age (<10 years old) [RR 0.34 (95% CI 0.25–0.44)].

## Discussion

The present study from Eastern India (with a developing country setting) provided the prevalence of metabolic syndrome in schoolchildren 6–16 years of age. The results were surprising in that the overall prevalence was high (21.8%), and around 11% of children ≤10 years fulfilled the MS criteria. The overall prevalence of overweight/obesity in the study population was 28.2%. Low HDL was found to be the most common single risk component of MS. Insulin resistance was higher in those with MS.

There have been various studies published worldwide on the prevalence of MS in children and adolescents (10, 14–25). These are mainly hospital-based studies, with few being conducted in school or community settings (10, 16, 19–21). Of the latter, only two previously published studies have provided the prevalence of MS in children ≤10 years of age (10, 16). Of these two studies, one found the prevalence of MS in children aged 6–11 years to be 21% if the mother had gestational diabetes mellitus (GDM) and to be 18% if the mother had no GDM (10). The other did not report the overall prevalence but reported the prevalence of individual risk factors in this age group in a general sample (not only in overweight/obese children) of schoolchildren (obesity−6.3%, elevated BP−10.4%, hypertriglyceridemia−6%, fasting hyperglycemia−2%, and HDL—not reported) (16). A systematic review published in 2013 analyzed data published from year 2003 and found the median prevalence of MS to be 3.3% (range 0–19.2%) in the general pediatric population, 11.9% (range 2.8–29.3%) in children who were overweight, and 29.2% (range 10–66%) in children who were obese (14).

The IDF criteria are most commonly used for the diagnosis of MS in the pediatric population (9). There are various other criteria available for defining pediatric MS that can falsely change the actual prevalence of MS (21). There is no standard definition for MS in children ≤10 year of age, and we used the definition published previously by Boney et al. on children in the USA (10). Boney et al. used BMI instead of waist circumference to define obesity. To our utter surprise, we found that 11% of children in this age group are fulfilling two or more criteria of MS in addition to obesity. There has been a dramatic change in the lifestyle of students with the adoption of western diet, less outdoor activity, more screen time activity, and increase in stress due to study pressure at school as well as at home. All these factors potentially increase the risk of developing obesity and MS (26).

We included children who were overweight and obese and compared the risk factors for MS. As expected, the individual risk factors were significantly more common in those with obesity. Overweight, obesity, and MS were more common in children from private schools than those from government schools. Though our objective was not to study the factors underlying this observation, a possible explanation includes differences in the socio-economic status and the lifestyle of children coming from schools private and those who were from government schools. Compared to boys, girls had a slightly increased prevalence of individual components/risk factors of MS except obesity, and the reason may be the female sex hormones. A history of a cardiovascular disease, diabetes, obesity, and hypertension in the family was present in a significant number of children in this study. This might have added to the increased prevalence of MS in the study population as shown in previous studies (14, 18, 25). Insulin resistance was more common in children with MS. This was also supported in the study by a higher prevalence of acanthosis nigricans in children with MS. A significant number of children also had acanthosis nigricans, reflected as an increase in insulin resistance as shown by increased HOMA-IR. Although a history of EBF for 6 months was present in 68.1%, we could not find a decreased risk of MS linked to EBF. Hepatomegaly was present in 8.1% of the children, which was not confirmed by ultrasonography to be of fatty origin but is likely to be due to it. This is an important finding as it may develop in the future to non-alcoholic steato-hepatitis, which is an important cause of chronic liver diseases in adults nowadays.

The definitions of MS in children have been extrapolated mostly from that of the adults, with the latter focusing on the central role of insulin in the development of MS (27, 28). In contrast to adults, there is no single definition of MS in pediatric literature (10, 16, 19–21). Barriers to this include adult cutoffs or a single set of cutoff points across all age groups, lack of normative data for plasma insulin level, pubertal insulin resistance, and lack of central obesity (waist) cutoff points in majority of the study settings. In the present study, children with hyper-insulinemia were found to have significantly higher values of other common cardiovascular risk factors (TG, BP, and WC). This has implications for the screening costs of children identified as overweight or obese. Early detection of hyper-insulinemia may indicate changes in metabolic profile before other more commonly measured cardiovascular risk factors are found outside the recommended ranges. However, it also has to be remembered that clustering of several metabolic indicators that are potentially of greater clinical significance would be ignored if hyper-insulinemia alone is used as a marker of MS in children (29).

An additional finding in our study was the high prevalence of central obesity (WC > 90th centile) even in those being overweight (as defined by BMI). More data on children are required on the morbidity-related definition of overweight using WC, which probably is a better predictor of metabolic risk than BMI (13, 16). A study on pre-pubertal children in the USA has suggested a single WC cutoff of 71 cm as a risk factor for adverse CVD risk factor profile, which may be true as the mean WC in the present study was too high (30).

The present study has some limitations. First, the use of western data cutoffs to define MS in children ≤10 years old may not truly reflect the prevalence in this age group. Second, we did not perform oral glucose tolerance test to define impaired glucose tolerance or type 2 diabetes mellitus. Third, we did not collect data regarding pubertal changes, physical activity, and dietary pattern. Finally, being a cross-sectional study, it does not provide information about causality.

## Conclusions

The present study found a higher prevalence of MS and insulin resistance in schoolchildren from Eastern India who were overweight/obese. The worsening epidemic of obesity and its associated long-term health risks highlight the need for a workable, consistent definition of MS in children to enable the investigation of prevalence and of both short- and long-term health outcomes.

## Data Availability Statement

All datasets generated for this study are included in the article/supplementary material.

## Ethics Statement

The studies involving human participants were reviewed and approved by the Institute Ethics Committee of AIIMS Bhubaneswar. Written informed consent/assent was collected from the children or the children's parents/guardians. All methods and experimental protocols in this study were conducted in accordance with the approved protocols and Ethics Committee's existing guidelines. Written informed consent to participate in this study was provided by the participants' legal guardian/next of kin.

## Author Contributions

RD conceived and designed the experiment. RD, MM, SP, and PR performed the experiment and analyzed the data. AS and SM provided comments and technical advice. RD, AS, SP, and SM wrote the manuscript. All the authors have discussed the results, commented on the manuscript, and agreed to be accountable for all aspects of the work in ensuring that questions related to the accuracy or integrity of any part of the work are appropriately investigated and resolved. RD will act as guarantor.

### Conflict of Interest

The authors declare that the research was conducted in the absence of any commercial or financial relationships that could be construed as a potential conflict of interest.
